# miR-26a-5p inhibits the proliferation of psoriasis-like keratinocytes *in vitro* and *in vivo* by dual interference with the CDC6/CCNE1 axis

**DOI:** 10.18632/aging.205618

**Published:** 2024-03-05

**Authors:** Jianing Li, Daxin Pang, Lin Zhou, Hongsheng Ouyang, Yaping Tian, Hao Yu

**Affiliations:** 1 Key Lab for Zoonoses Research, Ministry of Education, College of Animal Sciences, Jilin University, Changchun 130062, China; 2 Chongqing Research Institute, Jilin University, Chongqing 401123, China; 3 Chongqing Jitang Biotechnology Research Institute Co., Ltd., Chongqing 401123, China; 4 Joint International Research Laboratory of Reproduction and Development, School of Basic Medicine, Chong-qing Medical University, Chongqing 400016, China; 5 Department of Dermatology and Venerology, First Bethune Hospital of Jilin University, Changchun 130021, China

**Keywords:** psoriasis, miRNA, HaCaT cells, HEKs, miR-26a-5p, CDC6, CCNE1, imiquimod

## Abstract

Psoriasis is a chronic inflammatory proliferative dermatological ailment that currently lacks a definitive cure. Employing data mining techniques, this study identified a collection of substantially downregulated miRNAs (top 10). Notably, 32 targets were implicated in both the activation of the IL-17 signaling pathway and cell cycle dysregulation. *In silico* analysis revealed that one of these miRNAs, miR-26a-5p, is a highly conserved cross-species miRNA. Strikingly, the miR-26a-5p sequences in humans and mice are identical, and mmu-miR-26a-5p was found to target the same 7 cell cycle targets as its human counterpart, hsa-miR-26a-5p. Among these targets, CDC6 and CCNE1 were the most effective targets of miR-26a-5p, which was further validated *in vitro* using a dual luciferase reporter system and qPCR assay. The therapeutic assessment of miR-26a-5p revealed its remarkable efficacy in inhibiting the proliferation and G1/S transition of keratinocytes (HaCaT and HEKs) *in vitro*. *In vivo* experiments corroborated these findings, demonstrating that miR-26a-5p effectively suppressed imiquimod (IMQ)-induced psoriasis-like skin lesions in mice over an 8-day treatment period. Histological analysis via H&E staining revealed that miR-26a-5p treatment resulted in reduced keratinocyte thickness and immune cell infiltration into the spleens of IMQ-treated mice. Mechanistic investigations revealed that miR-26a-5p induced a cascade of downregulated genes associated with the IL-23/IL-17A axis, which is known to be critical in psoriasis pathogenesis, while concomitantly suppressing CDC6 and CCNE1 expression. These findings were corroborated by qPCR and Western blot analyses. Collectively, our study provides compelling evidence supporting the therapeutic potential of miR-26a-5p as a safe and reliable endogenous small nucleic acid for the treatment of psoriasis.

## INTRODUCTION

Psoriasis is a prevalent chronic, immune-mediated, inflammatory skin disease that seriously affects the quality of life of patients [1–4] and affects approximately 2–3% of the global population [5–7]. The pathogenesis of psoriasis involves complex interactions among T cells, dendritic cells, and various inflammatory cytokines [8, 9]. Plaque psoriasis (bullous pemphigoid) is the most common subtype of psoriasis, accounting for 80% of all cases. Currently, there is no definitive cure for psoriasis, and its occurrence is influenced by a complex interplay of genetic and environmental factors. Genetic susceptibility to psoriasis is primarily associated with genes involved in keratinocyte differentiation and inflammatory responses [10, 11].

Despite the prominent clinicopathological features of psoriasis, including a shortened cell cycle and hyperproliferation of keratinocytes, research has focused predominantly on the role of tumor necrosis factor-α (TNF-α) and the IL-23/IL-17A axis in driving disease pathogenesis [12]. Studies by Michael Woods et al. demonstrated that cyclin D1 and its kinase partners CDK4 and CDK6 can promote the proliferation of keratinocytes but not to a sufficient extent to overcome calcium-induced keratinocyte differentiation [13]. Research by Inohara et al. clearly revealed that the CDK complex regulates the cell cycle by phosphorylating the Rb protein. This complex represents a potential target of various growth factors and anti-growth factors in keratinocytes [14]. Cell division cycle 6 (CDC6) is a crucial regulator of DNA replication that has been proven to be upregulated in tumors and associated with cancer progression and prognosis [15, 16]. Shuna Sun et al. showed that berberine (BBR) can inhibit CDC6, leading to the downregulation of CDK4/6 and RB1 protein expression and ultimately inhibiting keratinocyte proliferation [17]. However, when a cell decides to divide, the level of CDC6 increases, and CDC6 reactivates the expression of Cyclin E genes (CCNE1 or CCNE2), especially CCNE1, which bind and activate CDK2 to coordinate cell cycle commitment and the G1/S transition [18, 19]. Ichihara et al. reported that the CCNE1 gene is overexpressed in the skin of psoriasis patients and can be disrupted by miR-424, thus inhibiting the proliferation of keratinocytes [20]. Additionally, IL-17A can promote the proliferation of epidermal keratinocytes by upregulating cell cycle-related genes such as CCNE1, CDCA5, and CDCA25A [21]. IL-17A alone is insufficient for a substantial inflammatory response and may cooperate with other cytokines (such as TNF-α, IL-23, IL-1β, IL-6, and IL-22) to amplify the proinflammatory cascade characteristic of psoriasis [22].

MicroRNAs (miRNAs) are a conserved class of endogenously expressed small noncoding RNAs (~22 nt) that are widely involved in fine-tuning various biological processes [23]. Cell cycle progression is closely regulated by miRNAs at every stage. For example, the let-7 family of miRNAs has been shown to control several regulatory factors of cell proliferation, including cyclin A2, cyclin B1, cyclin E2, CDK8, and possibly CDK1, among other cell cycle targets [24]. Since 2007, more than 250 miRNAs have been reported to be aberrantly expressed in psoriatic tissues, most of which were found in peripheral blood or psoriatic skin [25]. Only a few downregulated miRNAs, such as miR-21, miR-217, miR-194, miR-99a, miR-330, and miR-378a, have definite biological functions in the skin [26–31]. The specific roles of miRNAs in hyperproliferation, keratinocyte differentiation, apoptosis, and atypical immune activation in psoriasis have not been extensively investigated [32].

The primary objectives of this study were to utilize bioinformatics approaches to identify downregulated miRNAs in psoriasis patients and to assess the effects of reintroducing these downregulated miRNAs into psoriatic cells and tissues through *in vitro* and *in vivo* experiments. Specifically, we aimed to determine whether miRNA reintroduction contributes to psoriasis treatment by targeting key pathways and modulating disease-associated processes.

## MATERIALS AND METHODS

### Screening of DEGs and DEmiRs

To ensure the accuracy of the interaction between miRNAs and their targets, we selected the GSE142582 series, in which the miRNA profile and mRNA profile of each group came from the same sample. The human skin samples included in the GSE142582 series were obtained from psoriatic patients and healthy volunteers who received no drug treatment within three months of recruitment. Disease severity was assessed by the psoriatic area and severity index (PASI). Five-millimeter-thick perforated biopsies of psoriatic plaque tissue (PS) and five control skin samples were obtained from surgically discarded samples from healthy people (NNs) (provided by the Shanghai Tenth People’s Hospital) [33]. DEGs and differentially expressed miRNAs (DEmiRs) in the psoriasis lesion group (PS) and the normal skin group (NN) were analyzed using DESeq2 [34]. The screening criteria were set as FDR <0.1 and |log2-fold change (FC)| >1. Gene set enrichment analysis (GSEA) of the DEGs was performed via the WEB-based Gene Set Analysis Toolkit (http://www.webgestalt.org/) [35]. A *p*-value < 0.05 was considered to indicate statistical significance in screening the enrichment results. Genes enriched in psoriasis-related pathways were selected as candidate target genes for DEmiRs.

### Target gene prediction and miRNA-mRNA regulatory network construction

The DEmiR-associated targets of the dataset GSE142582 were predicted using TargetScan [36], miRTarBase [37], miRDB [38, 39], and RNAhybrid [40]. Only the targets included in all of these databases were selected for further analysis. The miRNA-target genes were subsequently overlapped with the DEGs, and the negative interaction pairs between the DEmiRs and DEGs were used to construct the miRNA–mRNA network via Cytoscape [41] software (version 3.9.1). RNAhybrid was further used to calculate the minimum free energies (MFEs) between miRNAs and target genes, whereas miRNA:mRNA hybridization was considered plausible when the MFE scores were in the relatively high-energy interval (−30 kcal/mol < MFE ≤ −14 kcal/mol) [42]. Hybridization was performed by using RNAhybrid in domain mode, and the short sequence was hybridized to the best fitting region of the long sequence. This tool is primarily meant to predict miRNA targets. The reporter assay validity for identifying microRNA-target interactions was performed with miRTarBase [43].

### Ethical statement

The animal experiments in this study were approved by the Animal Protection and Research Ethics Committee of Jilin University, and all procedures were performed strictly in accordance with the guidelines for the Care and Use of Laboratory Animals. Specific pathogen-free BALB/c mice were purchased from Liaoning Changsheng Biotechnology Co., Ltd., (Benxi, Liaoning, China), raised at the Experimental Animal Center of Jilin University, and fed a standard maintenance diet. All animal operations were performed under anesthesia, and every effort was made to minimize pain. The human tissue used for cell isolation was provided by the First Affiliated Hospital of Jilin University (2020-708), and informed consent and confidentiality were obtained from the donors.

### HaCaT cell culture

The human immortalized keratinocyte (HaCaT) cell line was purchased from American Type Culture Collection (ATCC-PCS-200-011). The cells were maintained in Dulbecco’s modified Eagle’s medium (DMEM; Gibco, Thermo Fisher Scientific, Waltham, MA, USA) supplemented with 10% (v/v) fetal bovine serum (FBS; Gibco) and 1% penicillin/streptomycin (Boster, Wuhan, China). The cells were cultivated in a humidified incubator at 37°C with 5% CO_2_. The medium was changed every two days.

### Isolation of human primary epidermal keratinocytes (HEKs)

Human skin tissue was obtained from the First Affiliated Hospital of Jilin University. After several washes with physiological saline containing antibiotics, the tissue was cut into small squares with a side length of 5 mm, soaked in 0.25% trypsin and incubated overnight at 4°C. After overnight incubation, the tissue fragments were transferred to phosphate-buffered saline (PBS) buffer supplemented with antibiotics, after which the epidermal layer was carefully removed using ophthalmic forceps. The peeled epidermal layer was cut into smaller pieces and centrifuged at 2000 rpm for 10 minutes. The supernatant was discarded after centrifugation, the epidermal fragments were resuspended in K-SFM (Cat. 17005042; Thermo Fisher Scientific) supplemented with 1% penicillin/streptomycin, and the mixture was placed in a 37°C incubator under 5% carbon dioxide.

### Induction of cells with TNF-α

Human recombinant tumor necrosis factor was purchased from PeproTech, Inc., (Cat. 300-1A, Rocky Hill, NJ, USA). The powder was initially reconstituted in water to 1.0 mg/mL, and for extended storage, the TNF-α solution was further diluted to the proper concentration using PBS containing 5% BSA and stored at −80°C. When used to induce cells, the TNF-α solution was further diluted with cell culture medium to the corresponding concentration, after which the cells were cultured for a certain duration.

### microRNA transfection

The microRNA and scramble-miRNA mimics were synthesized by GenScript (Nanjing, Jiangsu, China) and were dissolved in water to a concentration of 1 μg/mL for storage. The sequences are shown in Table 1. For starvation, the medium was changed to Opti-modified Eagle’s medium (Opti-MEM, Gibco, Thermo Fisher Scientific) 12 hours before transfection. Lipofectamine 3000 transfection reagent (Cat. L3000008, Invitrogen, Carlsbad, CA) was used for miRNA transfection, and the manufacturer’s protocol was followed. In brief, 5 μl of transfection reagent and 5 μg of miRNA were added to 125 μl of Opti-MEM, after which the diluted transfection reagent and miRNA mixture were mixed to a total volume of 250 μl. After resting for 15 min at room temperature, the mixtures were added to the cells in the six-well plate drop by drop. The same dose of transfection reagent without miRNAs was added to the control group. The basal medium was resuspended 8 h after transfection.

**Table 1:** Sequences of microRNAs.

**miRNA ID**	**Sequences (5′→3′)**
hsa-let-7a-5p	UGAGGUAGUAGGUUGUAUAGUU
hsa-let-7b-5p	UGAGGUAGUAGGUUGUGUGGUU
hsa-let-7c-5p	UGAGGUAGUAGGUUGUAUGGUU
hsa-let-7d-5p	AGAGGUAGUAGGUUGCAUAGUU
hsa-let-7g-3p	UGAGGUAGUAGUUUGUACAGUU
hsa-miR-22-3p	AAGCUGCCAGUUGAAGAACUGU
hsa-miR-26a-5p	UUCAAGUAAUCCAGGAUAGGCU
hsa-miR-30a-5p	UGUAAACAUCCUCGACUGGAAG
hsa-miR-195-5p	UAGCAGCACAGAAAUAUUGGC
hsa-miR-423-5p	UGAGGGGCAGAGAGCGAGACUUU
mmu-miR-26a-5p	UUCAAGUAAUCCAGGAUAGGCU
Scramble-miR-26a	GAUCGAUACCAUAGACUAUGGU

### Cell proliferation and viability assay

CCK-8 (Cat. AR1199, Beyotime, Shanghai, China) reagent was used to monitor cell viability. Cells (5,000 cells/well) were seeded into 96-well plates. The CCK-8 reagent was diluted 1:10 with DMEM, 100 μl was added to each well, and then, every group of cells was treated and incubated for 2 hours at 37°C. Optical density (OD) values were monitored using a microplate reader at 450 nm (Infinite M200 Pro NanoQuant, Tecan, Switzerland) every 12 hours. Cell viability curves were plotted with the OD values as the ordinate and points in time as the abscissa.

### Flow cytometric analysis

The cells were seeded into 6-well plates, cultured to 40–50% (5~6 × 10^5^) confluence, and then transfected with miRNAs for 72 h. The cells were harvested, washed with cold phosphate-buffered saline (PBS), and fixed in 70% ethanol at 4°C overnight. RNA was removed by using RNase A at 37°C for 30 min. Finally, the cells were stained with propidium iodide (PI; Cell Cycle and Apoptosis Analysis Kit, Cat. C1052; Beyotime, Shanghai, China) for 30 min at room temperature and analyzed on a FACSAria III flow cytometer (BD Biosciences). The cell cycle peak was generated using Modfit software (ver. 5.0).

### 5-Ethynyl-2′-deoxyuridine (EdU) staining

EdU staining was performed using a BeyoClick™ EdU Cell Proliferation Kit with Alexa Fluor 488 (Cat. C0071S; Beyotime, Shanghai, China) according to the manufacturer’s protocol. In brief, cells were plated in six-well plates after the corresponding treatments, incubated with 10 μM EdU for 2 hours at 37°C and then fixed with 4% paraformaldehyde for 15 min, permeabilized with 0.5% Triton X-100 for 20 min and stained with Click Additive Solution and Hoechst 33342 in the dark. Images were captured using an EVOS f1 fluorescence microscope (Thermo Fisher Scientific). The percentage of EdU-positive cells was analyzed using Image-Pro Plus 6.0 software (Media Cybernetics, Silver Spring, USA).

### IMQ-induced psoriatic mouse model

For establishment of IMQ-induced psoriasis-like skin inflammation, 20 six-week-old BALB/c female mice were randomly divided into four groups (one Vaseline group and three IMQ groups) after a one-week stabilization period. On the day before induction, the dorsal skin of the mice was shaved and then treated with 5% imiquimod cream (62.5 mg) (Sichuan Mingxin Pharmaceutical Co., Ltd., Chengdu, Sichuan, China), and the same dose of Vaseline was applied to the Vaseline group once daily for 1 to 4 days. The day before IMQ plastering was defined as Day 0, and the psoriatic mouse model was generated via continuous smearing for 4 days. The erythema score, scaling score, and skin thickness severity index were calculated from 1–5, accurate to one decimal place, and the three parameters were summed to obtain a cumulative score [44].

### Injection of miRNAs into a psoriatic mouse model

On the 4th day after the psoriasis model was established, the psoriatic mice were randomly divided into three groups: the IMQ group, the scr-miRNA (scrambled-microRNA) (GenScript, Nanjing, China) group, and the miR-26a group. The scr-miRNA group and the miR-26a (10 μg) group were subcutaneously injected with compounds from the Entranster™ *In Vivo* Kit (Cat. 18668-11-1; Engreen Biosystem Co., Ltd., Beijing, China) and scr-miRNA or miR-26a mimics, respectively. Moreover, the negative control group and IMQ group were injected with the abovementioned transfection reagent in combination with saline. Daily skin changes were recorded with photos by a Canon 700D Single lens Reflex camera.

### Histological analysis

The skin and spleen tissues from each group of mice were collected after euthanasia, soaked in 4% paraformaldehyde at 4°C overnight, dehydrated in increasing concentrations of ethanol for various durations (70%—6 h, 80%—1 h, 96%—1 h, 100%—3 h), and clarified in xylene, followed by embedding in paraffin. Sections 5 μm in thickness were cut, and after dewaxing, H&E staining was performed using an H&E staining kit (Cat. AR1180, Boster, Wuhan, Hubei, China). The stained sections were imaged with an Olympus CX43 microscope, and the layer thicknesses were measured by the tool within the software.

### Immunohistochemistry

Immunohistochemistry was performed by the standard procedure. In brief, the tissue slices were dewaxed in xylene for 10 minutes, after which the xylene was removed with PBS. The slices were placed in heated boiling EDTA antigen retrieval solution and boiled for 20 minutes. The slices were then cooled with cold water to room temperature, after which the antigen retrieval solution was removed with PBS. Normal goat serum solution (Cat. AR0009, Boster, Wuhan, Hubei, China) was added to the slices, which were incubated in a wet box for 30 minutes, after which the unbound serum protein was washed off with PBS. The primary antibody was diluted 100-fold and added to the slices. The samples were incubated in a wet box for 1–2 hours, after which the unbound primary antibody was washed off with PBS. The 100-fold diluted secondary antibody was added to the slices. The samples were incubated in a wet box for 1–2 hours. Finally, the unbound secondary antibody was washed off with PBS, the slices were dried, and the slides were observed under a microscope after they were dried. The antibodies used for the immunohistochemistry assays are shown in Table 2.

**Table 2:** Primary antibodies used in this study.

**Target**	**Host**	**Assay**	**Dilution**	**Manufacturer**	**Catalog Number**
CCNE1	Rabbit	WB	1:1000	BOSTER	A00543-2
		IHC	1:100		
CDC6	Rabbit	WB	1:1000	BOSTER	BA1726
		IHC	1:100		
GAPDH	Mouse	WB	1:2000	BOSTER	BM3876
IL-6	Rabbit	WB	1:1000	Wanleibio	WL02841
IL-17A	Rabbit	WB	1:1000	BOSTER	BA12416
IL-22	Rabbit	WB	1:1000	Wanleibio	WL04441
IL-23	Rabbit	WB	1:500	Wanleibio	WL01655
TNF-α	Rabbit	WB	1:1000	BOSTER	A00002-2
p-p65	Rabbit	WB	1:1000	Wanleibio	WL02169
p-STAT3	Rabbit	WB	1:1000	BOSTER	PB0540

Abbreviations: WB: Western blotting; IHC: Immunohistochemistry.

### Total RNA and miRNA extraction and qRT-PCR

For qRT-PCR analyses, total RNA was extracted from cells (72 hours after miRNA transfection) and tissues with TRIzol reagent (DP419, Tiangen, Beijing, China), and complementary DNA (cDNA) was synthesized using a FastKing RT Kit (with gDNase) (KR116, Tiangen Biotech, China) according to the manufacturer’s recommendations. A miRcute miRNA extraction kit (DP501, Tiangen, Beijing, China) was used for miRNA extraction, and a miRcute Plus miRNA First-Strand cDNA kit (KR211, Tiangen, Beijing, China) was used for miRNA RT-PCR. Real-time qPCR assays were performed using SuperReal PreMix Plus (SYBR Green) (FP205, Tiangen, Biotech, China), a miRcute Plus miRNA qPCR kit (FP411, Tiangen, Beijing, China), and an IQ5 Multicolor Real-Time PCR detection system (Bio-Rad, Hercules, California, USA). The expression levels of the treated samples were normalized to the levels of the GAPDH and U6 genes, and the relative gene expression levels were calculated via the 2^−ΔΔCT^ formula [45].

### Primer design and validation

The primer pairs used in the RT-qPCR assay were designed using AlleleID software version 7.0 (Premier Biosoft, http://www.premierbiosoft.com/). All the sequences were tested for their potential secondary structure and dimerization ability using the OligoAnalyzer 3.1 program (Integrated DNA Technologies, https://sg.idtdna.com/pages). The specificity of the primers was identified using the Primer-blast program (https://blast.ncbi.nlm.nih.gov/Blast.cgi). The validated sequences are shown in Tables 3, 4.

**Table 3:** Primers used in qPCR for the quantification of genes expression in skin tissues of mice.

**Gene ID**	**Sequences (5′→3′)**
*CCNE1*	Forward: AGGACTGCATTTCAGCCTCGGA
	Reverse: GAACTGCTCTCATCCTCGCCTG
*CCNE2*	Forward: GCCACCTGTACTGTCTGGAGGA
	Reverse: CTCCTGTGAACATGCCCAGCTT
*CDC6*	Forward: ACGTTCCTCCTCCGCTCAAAGA
	Reverse: GACTGCCAGCTTTCTTCCCACA
*CHEK1*	Forward: TGGCTTGGCAACGGTATTTCGG
	Reverse: TGGTCCCACGGCAATTCTCCA
*MAD2L1*	Forward: TCTGCGGTGAGGTTGGTAGTGT
	Reverse: CGACGGATAAATGCCACGCTGA
*PTTG1*	Forward: AGCCAGCAGAAAGGCTTTGGG
	Reverse: TGGGTAGGCATCATCAGGAGCA
*TTK*	Forward: AGCCTGATGATGCCCGTGACT
	Reverse: AGTTACGCATGGCCGTCTCCA
*STAT1*	Forward: TCACAGTGGTTCGAGCTTCAG
	Reverse: GCAAACGAGACATCATAGGCA
*STAT3*	Forward: CAATACCATTGACCTGCCGAT
	Reverse: GAGCGACTCAAACTGCCCT
*IL-6*	Forward: TTCCATCCAGTTGCCTTCTTG
	Reverse: GGGAGTGGTATCCTCTGTGAAGTC
*IL-17A*	Forward: CTCAGACTACCTCAACCGTTCC
	Reverse: ATGTGGTGGTCCAGCTTTCC
*IL-23A*	Forward: AATAATGTGCCCCGTATCCA
	Reverse: CATGGGGCTATCAGGGAGTA
*GAPDH*	Forward: AGGTCGGTGTGAACGGATTTG
	Reverse: TGTAGACCATGTAGTTGAGGTCA

**Table 4:** Primers used in qPCR for the quantification of miRNAs in skin tissues of mice.

**miRNA ID**	**Sequences (5′→3′)**
miR-26a-5p	Forward: GCAGTTCAAGTAATCCAGGATAG
	Reverse: GGTCCAGTTTTTTTTTTTTTTTAGC
U6	Forward: CTCGCTTCGGCAGCACA
	Reverse: CCTCCACAGCTTCAAGCTTTTG

### Protein isolation and western blotting

Total protein was extracted from tissues and cells using radioimmunoprecipitation assay lysis buffer (Cell lysis buffer for Western and IP; P0013; Beyotime, Shanghai, China) supplemented with phenylmethanesulfonyl fluoride (PMSF; ST506 Beyotime, Shanghai, China). The protein concentration was measured using an enhanced BCA protein assay kit (Cat. P0010, Beyotime, Shanghai, China). Total protein extracts were separated on 10% gels via SDS-PAGE and then transferred to 0.45 nm polyvinylidene fluoride membranes (Millipore, USA). The proteins were probed with specific antibodies after the blot was blocked with 5% nonfat milk (Cat. AR0104; Boster, Wuhan, China). The antibodies used are listed in Table 2.

### Dual-luciferase reporter assay

The target sites of miR-26a-5p on CDC6 and the CCNE1 3′UTR were predicted by mirPath v.3 from DIANA TOOLS (https://dianalab.e-ce.uth.gr/html/mirpathv3/index.php?r=mirpath). Dual-luciferase reporter plasmids containing a 200 bp fragment containing the target site of miR-26a-5p were synthesized by Sangon Biotech (Shanghai, China). HEK293 and NIH3T3 cells were transfected with 5 μg of Psi-CHECK2-wt CDC6-3′UTR (or wtCCNE1-3′UTR), 10 μmol of miR-26a and Psi-CHECK2-wt CDC6-3′UTR (or wtCCNE1-3′UTR); miR-26a and Psi-CHECK2-mut CDC6-3′UTR (or wtCCNE1-3′UTR); or Scr-miR-26a and Psi-CHECK2-wt CDC6-3′UTR (or wtCCNE1-3′UTR). Forty-eight hours after transfection, a Dual-Lumi™ II Luciferase Assay Kit (Cat. No. RG089S; Beyotime, Shanghai, China) and a microplate reader (Infinite M200 Pro NanoQuant, Tecan, Switzerland) were used to determine the fluorescence intensities of firefly luciferase and Renilla luciferase, the specific values of which were used as the relative luciferase activity.

### Statistical analysis

Statistical analyses were performed using GraphPad Prism for Windows software (GraphPad Software, version 8.0.1; San Diego, CA, USA; https://www.graphpad.com/). The values are expressed as the means ± SDs. Comparisons between two groups were performed using Student’s *t* test. ^*^p < 0.05, ^**^p < 0.01, ^***^p < 0.001, and ^****^p < 0.0001 are the significance thresholds, and ns means not significant.

### Availability of data and material

All data in the study are available.

## RESULTS

### *In silico* identification of the miR-26a-5p/CCNE1/CDC6 axis in psoriasis

**Figure 1:**
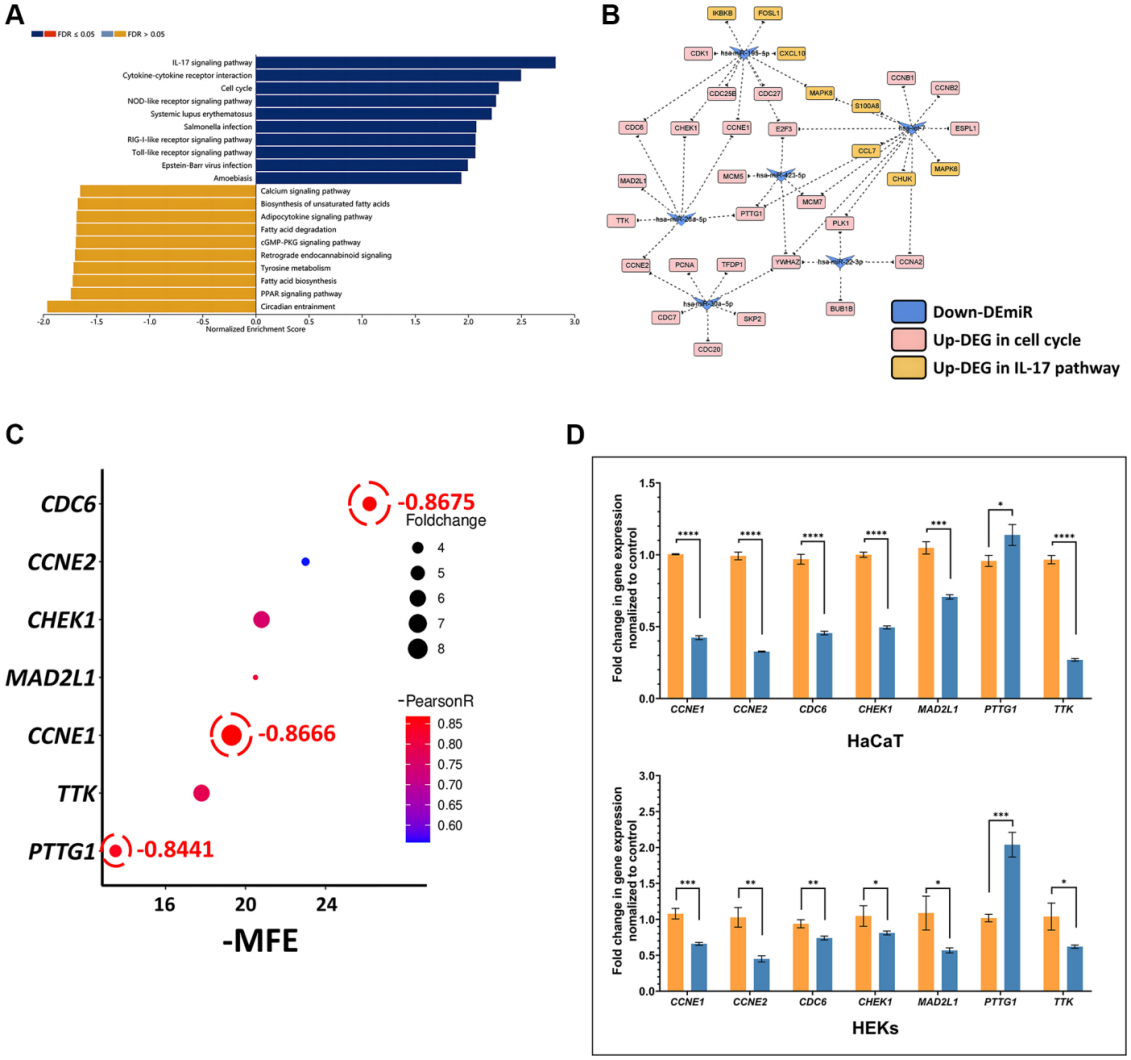
*In silico* identification of the miR-26a-5p/CCNE1/CDC6 axis in psoriasis (**A**) GSEA bar chart showing the top 10 enhanced and top 10 inhibited KEGG pathways for DEGs; the color scale represents the FDR, and the length of the bar represents the normalized enrichment score (NES) (positively correlated, blue >0; negatively correlated, orange <0). (**B**) The interaction network of 10 downregulated miRNAs and target genes involved in the cell cycle and the IL17 pathway. Let-7a-5p, let-7b-5p, let-7c-5p, let-7d-5p, and let-7g-3p are uniformly represented by let-7 because they all share the same target genes. (**C**) Multifactor correlation estimation between miR-26a-5p and target genes. (**D**) Target gene expression in HaCaT and HEKs cells. Orange columns: cells induced with TNF-α without miRNA transfection; blue columns: cells induced with TNF-α and transfected with miR-26a-5p. The data are presented as the mean ± SD (*n* = 3). ^*^p < 0.05; ^**^p < 0.01; ^***^p < 0.001; ^****^p < 0.0001.

We performed a gene set enrichment analysis (GSEA) of 2985 DEGs (1392 upregulated and 1593 downregulated DEGs) obtained from the GSE142582 dataset with the KEGG signaling pathways and found that the IL-17 signaling pathway and cell cycle were associated with the development of psoriasis among the pathways significantly enriched with upregulated genes (Figure 1A). We next selected the top 10 downregulated DEmiRs (let-7a-5p, let-7b-5p, let-7c-5p, let-7d-5p, let-7g-3p, miR-423-5p, miR-30a-5p, miR-26a-5p, miR-22-3p and miR-195-5p) from the 232 DEmiRs (99 upregulated and 133 downregulated DEmiRs) obtained from the GSE142582 dataset to analyze the targeting relationship between the upregulated genes in the two pathways, resulting in the generation of a miRNA-mRNA network based on 24 targets involved in the cell cycle and only 8 targets belonging to the IL-17 signaling pathway, as shown in Figure 1B. miR-26a-5p was first chosen as a candidate therapeutic miRNA. To identify reliable targets, we performed a comprehensive analysis considering the expression correlation, minimum free energy (MFE), and upregulated fold change values of the 7 target genes (CCNE1, CCNE2, CDC6, CHEK1, MAD2L1, PTTG1 and TTK). We found that CDC6, CCNE1 and PTTG1 exhibited the strongest negative correlation with miR-26a-5p (r < −0.8). However, PTTG1 had the lowest MFE (−13.5) in humans harboring miR-26a-5p, which suggested that PTTG1 may not be a primary target of miR-26a-5p in humans (Figure 1C). We therefore transfected miR-26a-5p mimics into TNF-α-induced HaCaT cells and human epidermal keratinocytes (HEKs). qPCR revealed that PTTG1 was not downregulated as was CDC6 or CCNE1 by miR-26a-5p, causing more than 50% downregulation of both HaCaT and HEK cell lines, suggesting that CDC6 and CCNE1 are the most reliable effective targets of miR-26a-5p (Figure 1D).

### Verification of cross-species conservation of the miR-26a-5/CDC6/CCNE1 axis in humans and mice

**Figure 2:**
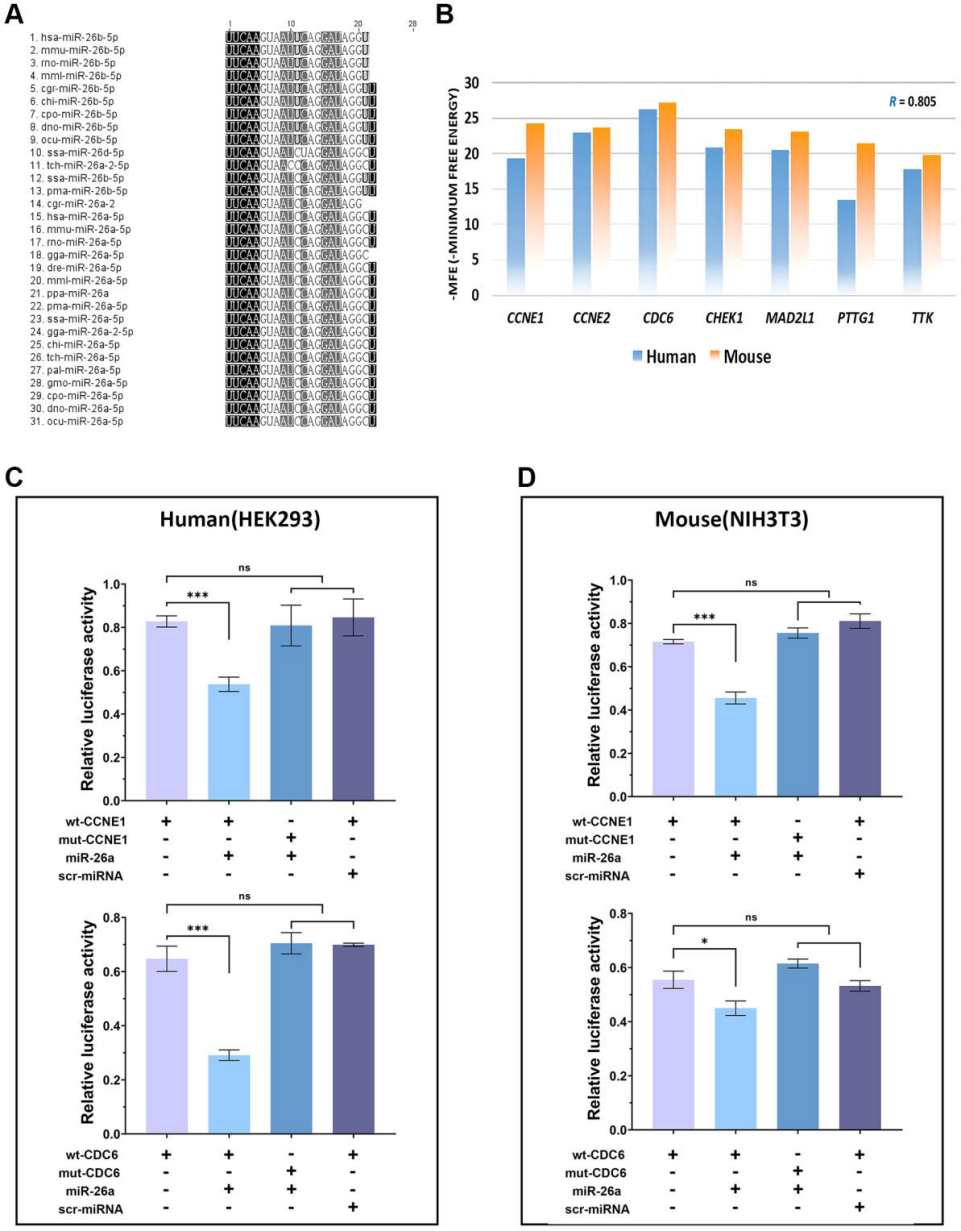
**Verification of cross-species conservation of the miR-26a-5/CDC6/CCNE1 axis.** (**A**) Multiple sequence alignment of orthologous miRNAs in the miR-26 family. (**B**) Comparison of the minimum free energy of miR-26a-5p at the 3′UTR binding sites of target genes between humans (blue columns) and mice (orange columns). (**C**, **D**) Dual-luciferase reporter assay of target genes of miR-26a-5p and statistical analysis of the relative fluorescence intensity of each transfection group. The data are presented as the mean ± SD (*n* = 3). ^*^p < 0.05. ^***^p < 0.001.

To ensure consistency between our *in vivo* and *in vitro* experiments, we first performed multiple sequence alignment of the published miR-26 family sequences. The results showed that only miR-26 showed cross-species conservation, and all 29 miR-26 sequences were the same except for those of ssa (*Salmo salar*) and tch (*Tupaia chinensis*) (Figure 2A). Fortunately, the relationships of 7 targets (CCNE1, CCNE2, CDC6, CHEK1, MAD2L1, PTTG1 and TTK) of miR-26a-5p were confirmed in mice with target prediction analysis, and CDC6 was the gene that most highly bound to miR-26a-5p in both species (Figure 2B). To confirm our hypothesis, we performed dual luciferase analysis to verify that CDC6 and CCNE1 are direct targets of miR-26a-5p in both humans and mice. ELISAs also revealed that the fluorescence intensity of cell extracts from 3′UTR- and miR-26a-cotransfected cells was significantly lower than that of the cells from the mutant sequence-cotransfected and scr-miR-transfected groups by more than 30% (*p* < 0.001). These findings demonstrated that miR-26a-5p can disrupt the expression of CDC6 and CCNE1 in both human and mouse cells, confirming their direct interaction and regulatory relationship (Figure 2C, 2D).

### *In vitro* validation of the role of miR-26a-5p in inhibiting keratinocyte proliferation

**Figure 3:**
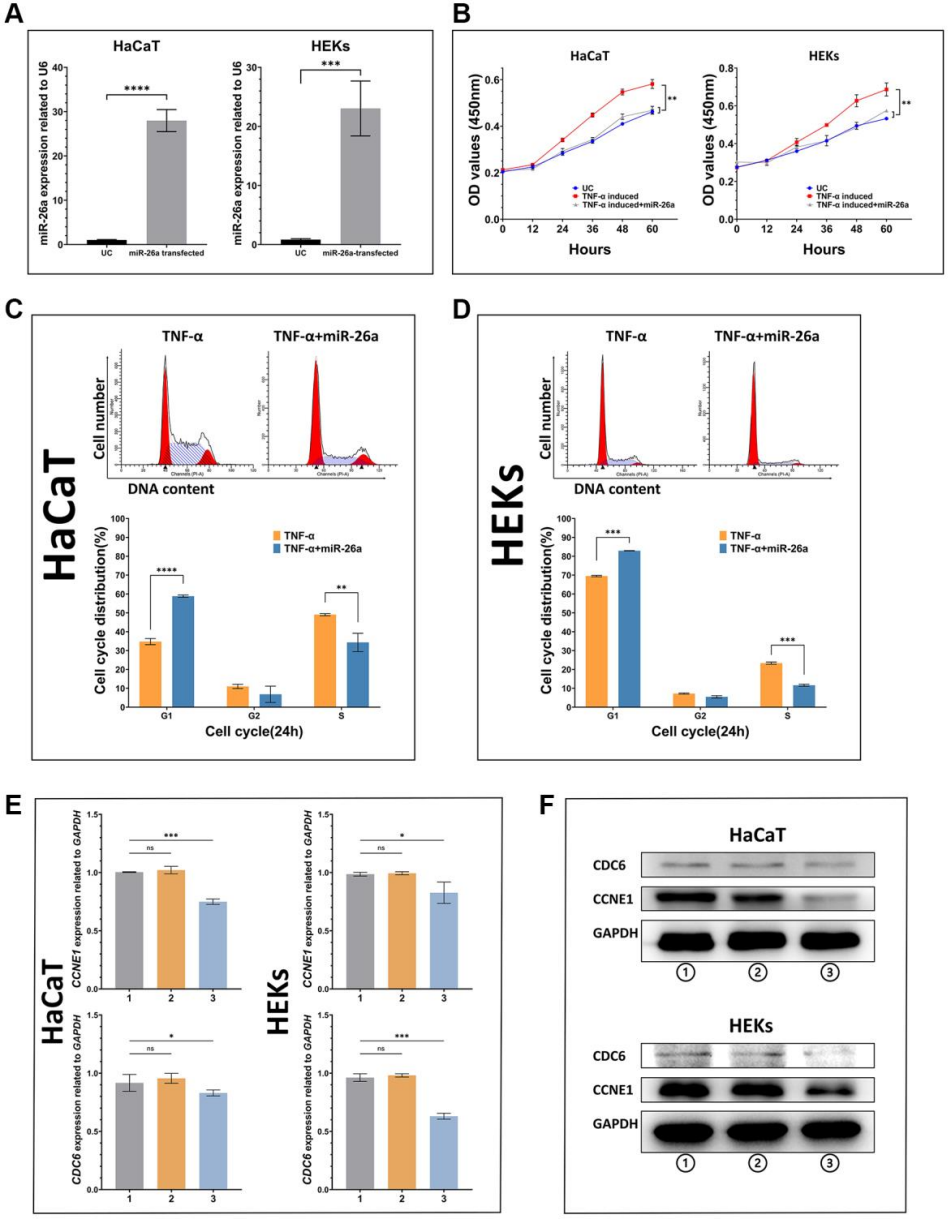
*In vitro* validation of the ability of miR-26a-5p to inhibit keratinocyte proliferation (**A**) miR-26a-5p expression in transfected HaCaT cells and HEKs compared with that in untreated control cells. (**B**) Proliferative ability (growth curve) of cells in each group. Dark blue line: untreated control; red line: cells induced with TNF-α; gray line: cells induced with TNF-α and transfected with miR-26a-5p. (**C**, **D**) Proportional changes in the cell cycle according to flow cytometry assays between TNF-α-induced cells transfected with or without miR-26a-5p. (**E**, **F**) mRNA and protein expression of miR-26a targets in HaCaT cells and HEKs. Group 1: Nontreated cells; Group 2: Cells with transfection reagent added; Group 3: Cells transfected with miR-26a. The data are presented as the mean ± SD (*n* = 5). ^*^p < 0.05; ^**^p < 0.01; ^***^p < 0.001; ^****^p < 0.0001.

**Figure 4:**
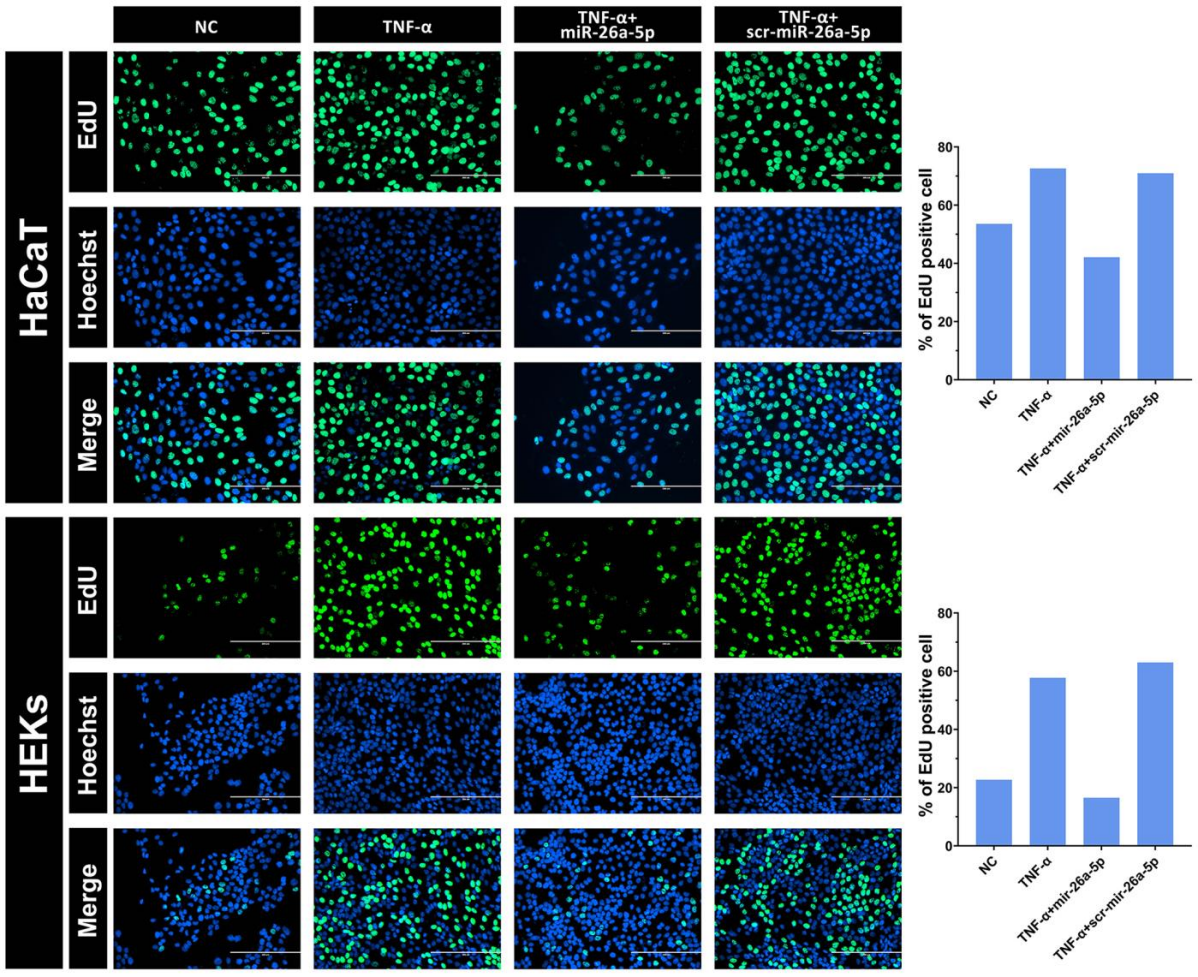
**Intuitive observation of the cell proliferation rate of HaCaT keratinocytes and HEKs by EdU staining.** After 48 hours, the cells were transfected with 50 nM miRNA mimics. The percentage of EdU incorporation was analyzed using Image-Pro Plus 6.0 software. Scale bars: 200 μm.

To investigate the inhibitory effect of miR-26a-5p on the proliferation of keratinocytes under pathological and normal physiological conditions, we selected HaCaT cells and HEKs as the target cells for miRNA transfection, and miR-26a-5p was measured in the transfected cells by qRT-PCR (Figure 3A). The viability assays (CCK-8) of both miR-26a-5p mimic-transfected HaCaT cells and HEKs showed significantly decreased proliferation compared to that of the negative control cells (Figure 3B). Furthermore, according to the EdU incorporation assays, the number of EdU-positive cells was significantly reduced following the above transfection of miR-26a-5p mimics in both cell types. Interestingly, we found that the number of HaCaT cells was significantly lower than that of HEKs after transfection with the miR-26a-5p mimics, indicating that miR-26a-5p seems to have a stronger "killing" effect on HaCaT cells (Figure 4). We further observed that miR-26a-5p induced cell cycle arrest at the G1/S phase in HaCaT cells and HEKs by a flow cytometry assay. As shown in Figure 3C, 3D, following treatment with miR-26a-5p, a greater percentage of cells remained in the G0/G1 phase of the cell cycle (HaCaT: 34% to 59%; HEKs: 69% to 83%), and this change was accompanied by a reduced percentage of cells in the S phase (HaCaT: 49% to 34%; HEKs: 23% to 11%). In addition, we found that miR-26a-5p significantly reduced the expression of *CCNE1* and *CDC6* at the mRNA level (Figure 3E). The western blot results further confirmed that miR-26a-5p, as predicted by the luciferase reporter gene assay, was able to interfere with CDC6 and CCNE1 expression *in vitro* (Figure 3F) Finally, to ensure the safety of miR-26a-5p for future clinical treatment, researchers must evaluate the effect of the dosage of miR-26a-5p on the proliferation of normal cells. Therefore, we performed a proliferation inhibition assay on uninduced HaCaT and HEK cells, and the CCK-8 results showed that 2, 5 and 8 μg of miR-26a-5p did not affect the viability of HaCaT or HEK cells that were not induced by TNF-a (Supplementary Figure 1).

### *In vivo* evaluation of the therapeutic effects of miR-26a-5p in a psoriasis mouse model

**Figure 5:**
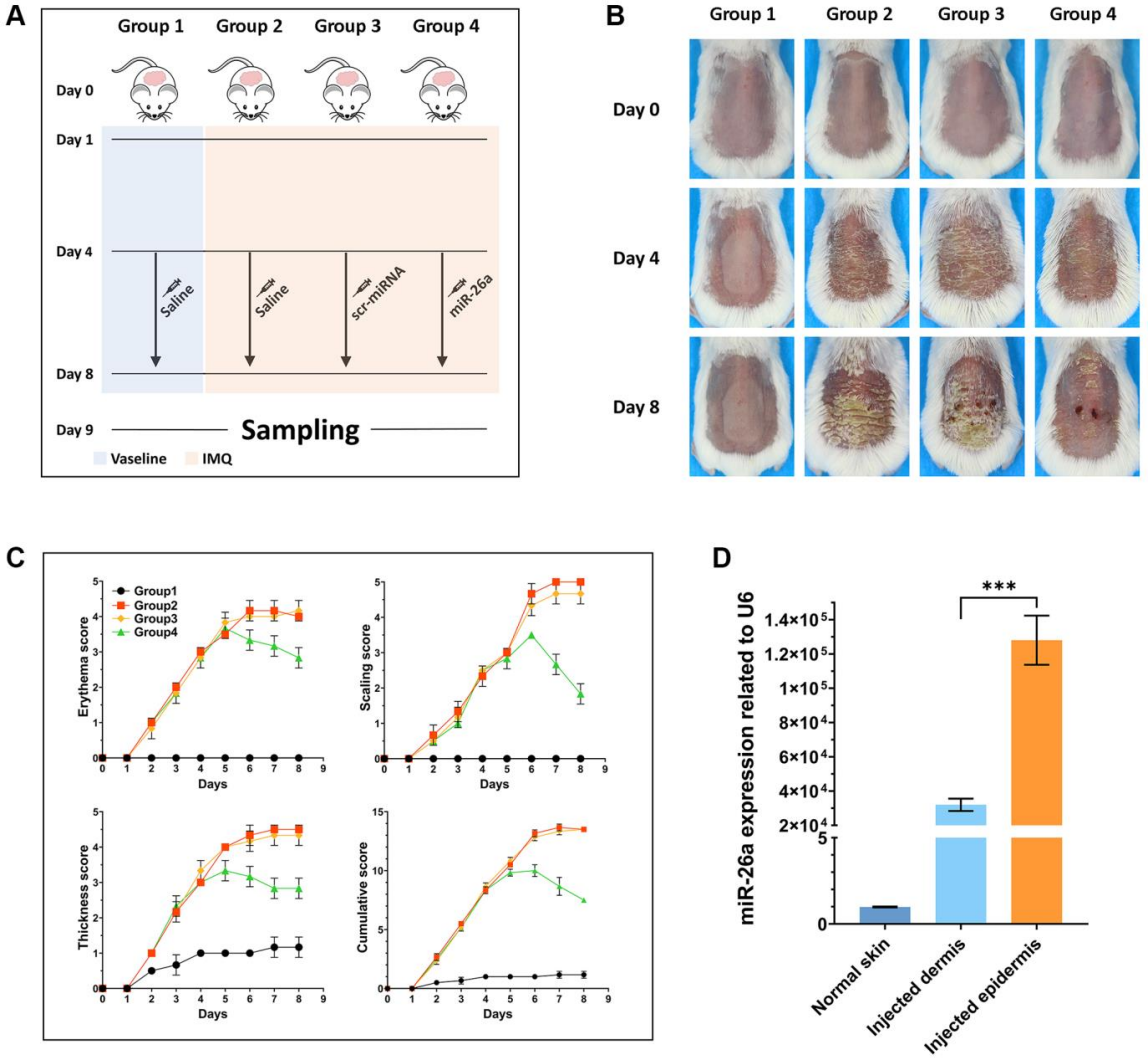
***In vivo* study design and pathological observation of mouse skin tissue.** (**A**) Schematic diagram of the whole treatment process for the mice in each group. In brief, the hair on the backs of all the mice was shaved off on Day 0. From Day 1 to Day 8, IMQ was administered to the backs of the mice in Groups 2, 3 and 4. Moreover, the backs of the mice in Group 1 were daubed with Vaseline. From Day 4 to Day 8, the mice in Groups 1 and 2 were injected with saline once every day, and the mice in Groups 3 and 4 were injected with scr-miRNA and the miR-26a-5p mimic, respectively. Tissue samples were collected on Day 9. (**B**) Representative macroscopic views of the dorsal skin of mice in each group. (**C**) Statistical analysis of the skin erythema score, scale score, and skin thickness severity index of each group of mice. (**D**) miR-26a expression in the dermis and epidermis of injected mice and in the normal skin tissue of mice in the Vaseline group. The data are presented as the mean ± SD (*n* = 5). ^***^p < 0.001.

**Figure 6:**
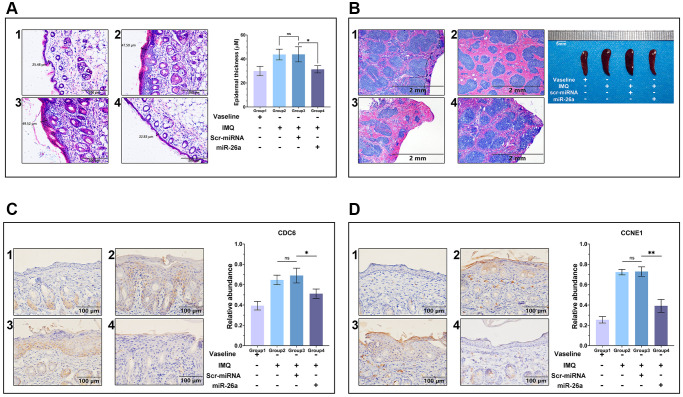
**Histology and immunohistology of skin tissue and spleen sections.** (**A**) H&E staining of paraffin sections of the dorsal skin and comparison of epidermal layer thickness. Scale bar: 200 μm. (**B**) Spleen sections and circumferential comparisons of mice from each group. Scale bar: 2 mm. (**C**, **D**) CCNE1 and CDC6 antibody staining of skin tissue sections for immunohistology analysis. Scale bar: 100 μm. The data are presented as the mean ± SD (*n* = 5). ^*^p < 0.05; ^**^p < 0.01.

To further assess the efficacy of miR-26a-5p in the treatment of psoriasis *in vivo*, we topically applied imiquimod cream containing 5% IMQ to the shaved back skin of the mice for eight consecutive days to induce psoriasis. The mice in the Vaseline group and IMQ group were injected with saline (0.9%) once a day, while those in the miRNA treatment group were injected intradermally with miR-26a-5p and scr-miRNA from 4 to 8 days (Figure 5A). Compared with those in the IMQ-treated wild-type (WT) group and scr-miRNA-injected group, the erythema score, scaling score, and skin thickness severity index were markedly lower in the miR-26a-5p treatment group (Figure 5B, 5C). Then, we isolated the epidermis and dermis from miRNA-injected skin samples, and the qRT-qPCR results showed that the content of miR-26a-5p in the epidermis was much greater than that in the dermis (Figure 5D). Then, the dorsal skin samples of each group were stained with hematoxylin and eosin (H&E), and the thickness of the epidermis was measured. Quantitative analysis revealed that the thickness of the epidermis was greatest in the IMQ and SCR-miR groups and was significantly greater than that in the miR (miR-26a-5p) and Vaseline (control) groups. As observed above, the thickness of the epidermis of the mice treated with miR-26a-5p was very similar to that of the control group (Figure 6A). Moreover, spleen sections from the miR-26a-5p group showed no white pulp depletion (Figure 6B). The white pulp and red pulp boundaries were also clearer in the Vaseline group and the miR-26a-5p group than in the IMQ and scr-miR groups, indicating that miR-26a-5p not only inhibited the proliferation of epidermal keratinocytes in psoriasis-like skin but also reduced the inflammatory reaction in the spleen. Changes in the spleen circumference were also observed, suggesting that the spleen was enlarged due to the inflammatory reaction. The above results confirmed that the *in vivo* therapeutic effects of miR-26a-5p in the IMQ mouse model are consistent with those in patients with psoriasis. Furthermore, we found that normal mouse dorsal skin also exhibited tolerance to 10 μg of miR-26a-5p (Supplementary Figure 2). Finally, we confirmed that miR-26a-5p triggered cascade downregulation of biomarkers from the IL-23/IL-17A axis associated with psoriasis development while suppressing CDC6 and CCNE1 expression (Figure 6C, 6D). These findings suggested that miR-26a-5p-induced cell cycle arrest can in turn weaken the activity of driving factors such as IL-17A.

**Figure 7:**
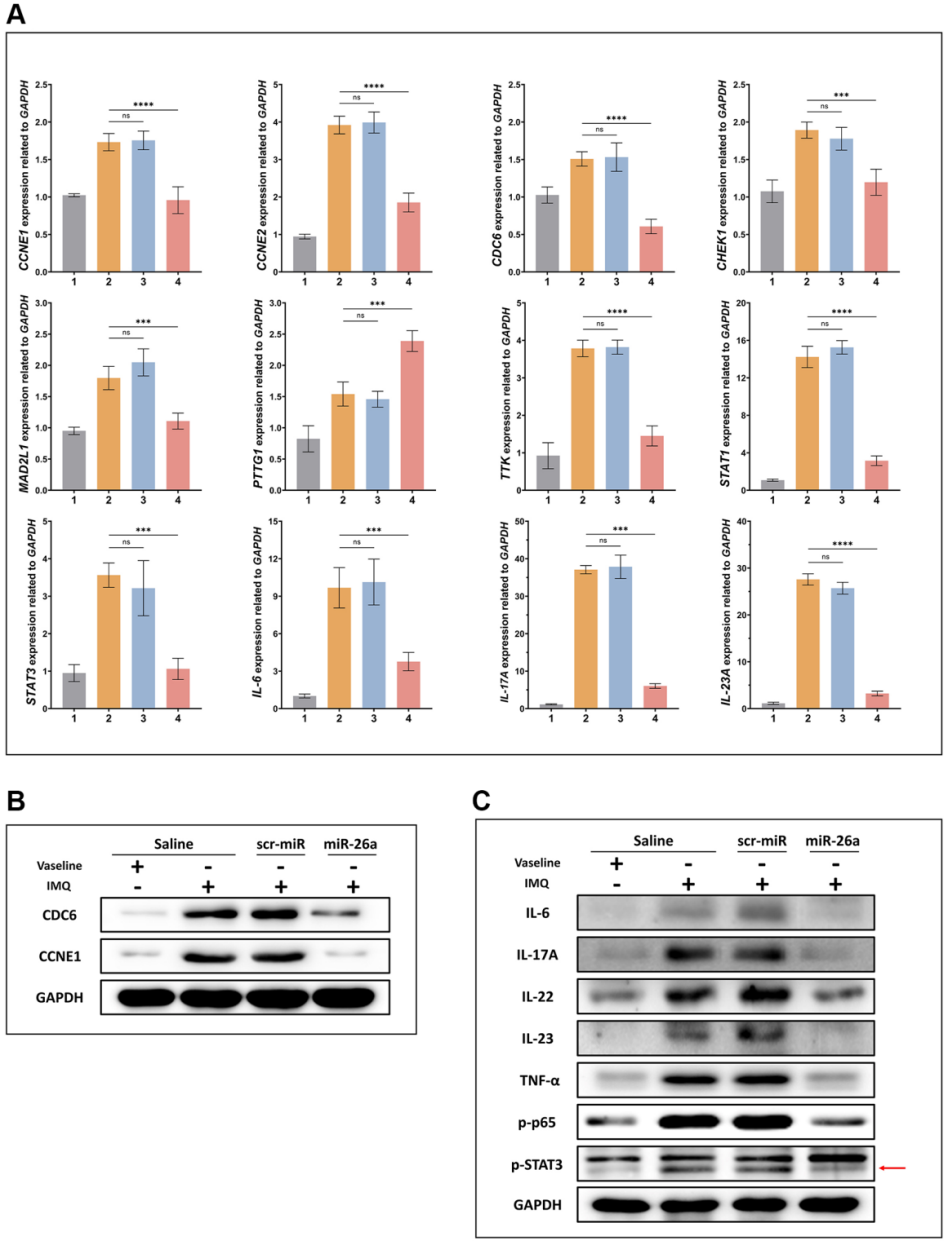
**Expression of proteins and genes related to psoriasis.** (**A**) mRNA expression of CDC6, CCNE1, and representative genes of the IL-23/IL-17A/STAT3 axis was detected in the IMQ-induced mouse model using qRT-PCR. (**B**, **C**) The protein expression of CDC6, CCNE1, and representative genes of the IL-23/IL-17A/STAT3 axis was examined in the IMQ-induced mouse model using Western blotting. The data are presented as the mean ± SD (*n* = 5). ^*^p < 0.05. ^**^p < 0.01. ^***^p < 0.001. ^****^p < 0.0001.

Based on these experimental results, 4 days after miR-26a-5p was injected subcutaneously into IMQ-induced mice, PTTG1 was not downregulated at the mRNA level among the 7 target genes (Figure 7A). CDC6 and CCNE1, which act as initial DNA replication switches, were downregulated at the protein level (Figure 7B). In addition, the core genes in the IL-23/IL-17A axis were downregulated by miR-26a-5p interference (Figure 7C).

## DISCUSSION

Psoriasis is an inflammatory skin disease characterized by a proliferative epidermis and is characterized mainly by epidermal hyperplasia [46]. The regulation of gene expression by miRNAs at the post-transcriptional level is considered to be an important epigenetic mechanism that appears to be involved in inflammation and keratinocyte proliferation or apoptosis in psoriasis [47]. In this study, we investigated the use of a safe small RNA drug that can be used to treat psoriasis; this drug result in no immune rejection and is easy to synthesize. It is also necessary to elucidate the molecular mechanism through which this drug inhibits the proliferation of epidermal keratinocytes. Our *in vitro* results clearly demonstrated the safety and efficacy of miR-26a-5p as an endogenous small RNA for inhibiting keratinocyte proliferation. Moreover, the analysis and *in vivo* validation of the consistency of the miR-26a-5p sequence and targets between humans and mice confirmed that the results from the IMQ-induced mouse model support the authenticity of the clinical trial (human).

In recent decades, the Th17 pathway has been regarded as the main pathogenic mode of psoriasis. Clinical studies have shown that IL-23, IL-17, and TNF-α play central roles in driving disease pathology [48–50]. The inhibition of local immune amplification has dominated the field of psoriasis treatment strategies [51]. Unfortunately, the cell cycle has never been able to become an attractive and alternative therapeutic option.

The psoriatic epidermis is much thicker than the normal epidermis and contains a greatly increased number of proliferating or germinating cells located in the lower epidermis. Furthermore, the germinal cells move upward and pass through the thicker psoriatic epidermis much faster. Weinstein et al. revealed 50 years ago that the normal epidermal cell cycle length was 457 hours and that the psoriatic epidermal cell cycle length was 37.5 hours. [52, 53]. These two cell cycle durations help explain the clinically rising scales in psoriasis and the principle of treating psoriasis with cancer chemotherapy drugs such as methotrexate [54, 55]. The precise transition from the G1 to S phase in the cell cycle is essential for controlling eukaryotic cell proliferation. Dysregulation of this transition promotes the development of excessive cell proliferation, such as oncogenesis [56]. In the G1 phase, growth-dependent cyclin-dependent kinase (CDK) activity promotes DNA replication and initiates the G1–S phase transition. CDK activation initiates a positive feedback loop, which further increases CDK activity, leading to cell division by inducing genome-wide transcriptional changes. G1–S transcripts encode proteins that regulate downstream cell cycle events [57]. Mutations in the retinoblastoma tumor suppressor gene (RB) or in components regulating the CDK-RB-E2F pathway have been recognized in almost all human malignancies. Therefore, re-establishing cell cycle control through cell cycle protein-dependent kinase (CDK) inhibition has become an attractive option in the development of targeted cancer therapies. Extensive studies suggest that the CDK-RB-E2F pathway is essential for the control of cell proliferation [58].

**Figure 8:**
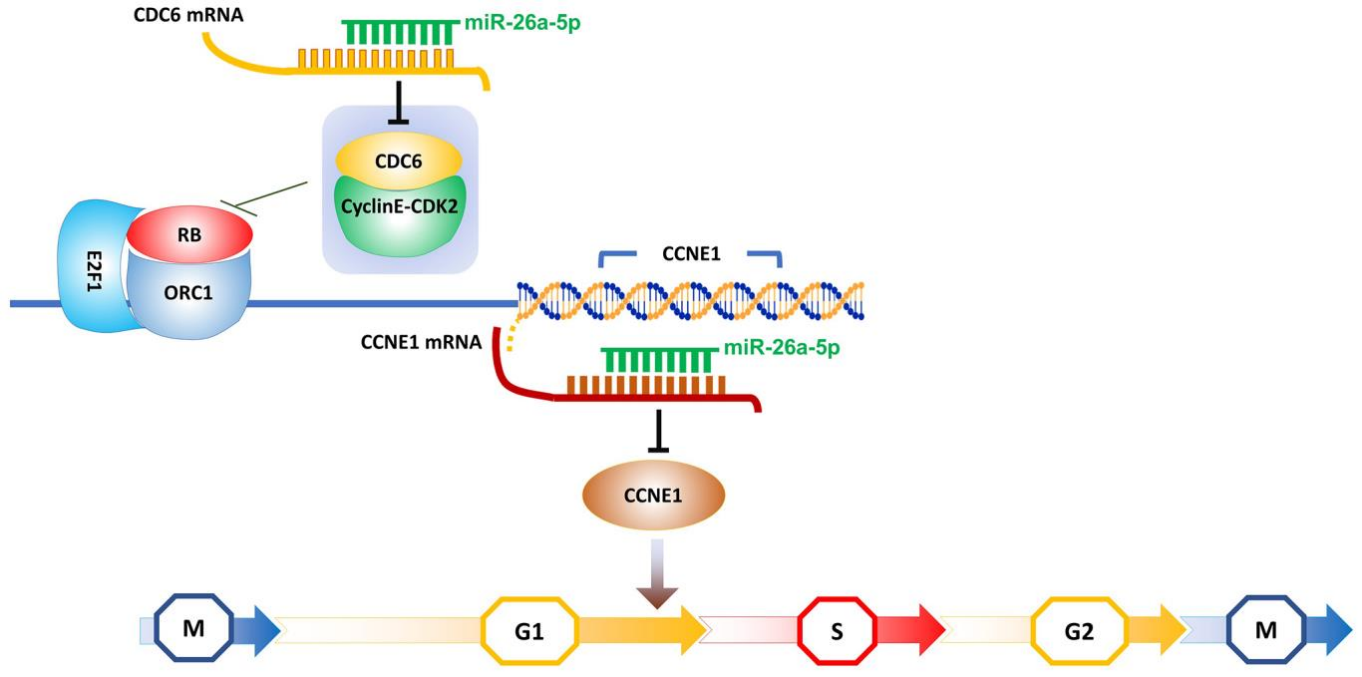
Schematic showing the hypothesized model of miR-26a-5p double interference on CCNE1 transcription.

RB is an effective inhibitory factor of G1-S transcription [59], and its discovery more than 20 years ago first suggested the dependency of cell cycle commitment on transcriptional regulation in G1 [60, 61]. Subsequent studies confirmed that the broad mechanisms of eukaryotic G1 cell cycle control are highly conserved [62]. Prior to the initiation of DNA replication, many proteins must assemble at multiple locations on the DNA molecule to form what is known as the pre-replication complex. One of these components is the ORC1 protein, which recruits another protein called CDC6. Hossain and Stillman reported that the combination of CDC6 and cyclin E-CDK2 can abolish the interaction between ORC1 and RB [63]. This event likely contributes to the relief of ORC1-mediated repression of the cyclin E (CCNE1) gene, resulting in a dramatic increase in CCNE1 production and subsequently promoting the next round of DNA replication and division [64]. Transcription of CCNE1 is known to be regulated by the E2F1 transcription factor and repressed by the RB protein [65]. The opposite effects of ORC1 and CDC6 on cyclin E levels ensure the stability of the genome. However, in recent years, the increased expression of CCNE1 in psoriasis has been considered a direct contribution of IL17A, while the increased expression of CDC6 is indirectly caused by the increased phosphorylation of STAT3 mediated by RIP4, which is a result of the interplay between IL17A and STAT3 [21, 66]. To date, multiple studies have demonstrated that suppressing the proliferation of keratinocytes by blocking the initiation of DNA replication via inhibitors is feasible. For example, TGF-β can inhibit the binding of cyclin E to CDK2, thus blocking the shift of cells from the G1 phase to the S phase in a human keratinocyte cell line [67]. Therefore, based on the above DNA replication initiation model, we propose that miR-26a-5p blocks the transition of keratinocytes from the G1 phase to the S phase by dual interference with the transcription of CCNE1. In the pretranscriptional stage of CCNE1, miR-26a-5p could downregulate the mRNA expression of CDC6 to weaken the synergistic effect of the CDC6 protein and cyclin E-CDK2 on the removal of RB, thus allowing ORC1 to act as a repressor protein to inhibit CCNE1 transcription. Moreover, miR-26a-5p can reduce the synthesis of the CCNE1 protein by directly silencing its mRNA at the post-transcriptional level (Figure 8). By flow cytometry analysis of HaCaT cells and HEKs, it was found that the proportion of G1 phase cells increased after treatment with miR-26a-5p. Moreover, the percentage of cells in the S phase decreased (Figure 3C, 3D), thus confirming that miR-26a-5p blocked the G1-to-S phase transition.

The innate properties of miRNAs make them very attractive potential biomarkers [68–70]. Our main rationale for preferring miR-26a-5p among the 10 candidate miRNA candidates is that the cross-species conservation of miR-26a-5p guarantees that the therapeutic efficacy of this miRNA in IMQ-induced mouse models is consistent with that in patients with psoriasis. However, the off-target effects of small RNAs can cause very unpredictable side effects on nontarget cells or tissues. Fortunately, miR-26a-5p exhibited a reliable safety profile in our series of experiments. Unlike internal organs, psoriasis can be treated by administering or injecting the drug precisely into the patient’s skin, substantially reducing damage to nontargeted cells. The results of our toxicity assay on normal cells suggested that cell proliferation is more sensitive to a low abundance of miR-26a-5p, which also largely reduces our concern that miR-26a-5p interferes with the safe growth of normal cells adjacent to skin lesion tissues; thus, we favor miR-26a-5p as a candidate small RNA with therapeutic potential for psoriasis. To date, cellular protection against the overexpression of miRNAs is complex and intractable. Jun et al. revealed a large number of potential lncRNAs, such as MALAT1, SNHG6 and OTUD6B, that can take up miR-26a-5p to regulate tumor cell proliferation, invasion and metastasis through the construction of a competitive endogenous RNA (ceRNA) interaction network [71]. However, our *in vivo* and *in vitro* experiments did not reveal that miR-26a-5p inhibited the proliferation of normal cells. If accurate quantification is not possible, subcutaneous injection of small RNAs is a potential threat to healthy tissue adjacent to the lesion. At present, the best solution is transdermal drug delivery. The use of hydrophobic cations encapsulating siRNA and choline-giant acid ionic liquids (CAGE) to enhance skin permeability has been proven to be feasible in porcine skin [72]. As suggested by Pankaj Dwivedi et al., understanding the potential signal transduction mechanism is necessary to identify targets for treating immune diseases [73]. Interestingly, driven by evolution, the genomic sites of miR-26a and miR-26b were located within the introns of genes encoding proteins of the carboxy-terminal domain RNA polymerase II polypeptide A small phosphatase (CTDSP) family. miR-26a/b and its host gene were found to synergistically activate the pRb protein and block the G1-S phase transition [74]. Furthermore, we reconfirmed the targeting relationships by dual-luciferase reporter assays. Additionally, Trohatou et al. verified that miR-26a mediates adipogenesis of amniotic fluid mesenchymal stem cells by interfering with CCNE1 [75], and Xin Zhang et al. demonstrated that miR-26a/b can regulate DNA replication licensing, tumorigenesis, and prognosis by targeting CDC6 in lung cancer [33]. Interestingly, miR-26a was recently reported to reduce the expression of CDC6 by binding to the lncRNA NORAD (noncoding RNA activated by DNA damage), thereby inhibiting the proliferation of keratinocytes, which further enhances the development potential of miR-26a-5p as a therapeutic small nucleic acid drug [76]. We propose that dual interference of miR-26a-5p in the CDC6/CCNE1 axis may be a universal mechanism for inhibiting the proliferation of cells. Previous studies have suggested that miR-26a-5p may function as a tumor suppressor in many cancers. For example, miR-26a-5p is reduced in hepatocellular carcinoma (HCC) and can inhibit tumor angiogenesis through HGF-cMet signaling [77]. In addition, in breast cancer, the expression of miR-26a-5p is reduced, and the overexpression of this miRNA leads to the inhibition of tumor growth and metastasis [78, 79]. In contrast, miR-26a-5p was found to serve as an oncogenic miRNA in non-small cell lung cancer by targeting FAF1 [80]. Interestingly, in our study, where the two targets with the highest correlation with miR-26a-5p, CDC6, and CCNE1 were also found to be proto-oncogenes [81–83], we confirmed via GEPIA (http://gepia.cancer-pku.cn/) that CDC6 and CCNE1 are highly expressed in most known tumors, which provides a breakthrough in our understanding of psoriasis treatment with anticancer drugs (Figure 6C).

Moreover, we speculate that miR-26a-5p interferes not only with the overexpression of CDC6 and CCNE but also with the normal expression of RB. Rb1, CCNE2 and IL6 were also found in the miR-26a-5p target list provided by miRTarBase, indicating that the CDC6/CCNE1 axis harbors the core targets of miR-26a-5p. We also retained several potential multitarget miRNAs for later studies, such as miR-195-5p, which, like miR-26a-5p, also interferes with CDC6 and CCNE1 (Figure 8), as demonstrated in oral squamous cell carcinoma cell lines [84].

## CONCLUSION

In summary, this study identified 10 potential therapeutic miRNAs from public transcriptome data using bioinformatics approaches. Among them, miR-26a-5p was first verified to suppress the proliferation of keratinocytes both *in vitro* and *in vivo*. Our findings support the hypothesis that dual interference of miR-26a-5p on CCNE1 transcription disrupts the initiation of DNA replication via the CDK-RB-E2F pathway. Our work may facilitate the development of small nucleic acid drugs for psoriasis treatment in the future.

## Supplementary Materials

Supplementary Figures
