# Supplementary material for: miR-26a-5p inhibits the proliferation of psoriasis-like keratinocytes in vitro and in vivo by dual interference with the CDC6/CCNE1 axis
Source: Aging (Albany NY). 2024 Mar 5;16(5):4631–53. doi: 10.18632/aging.205618 (PMC10968694; doi:10.18632/aging.205618)
Supplement: Supplementary Figures [file aging-16-205618-s001.pdf]

SUPPLEMENTARY FIGURES

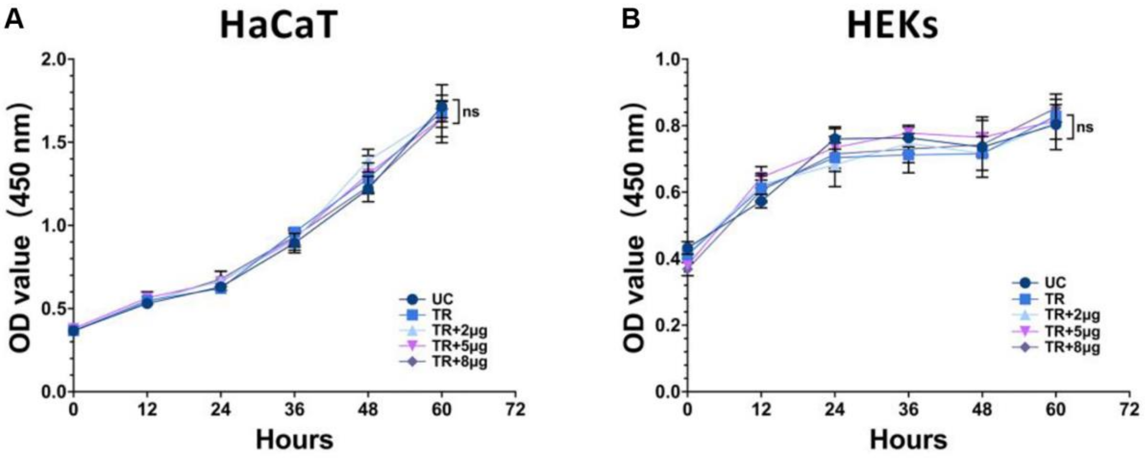

Supplementary Figure 1. Comparison of proliferative ability of HaCaT cells (**A**) and HEKs (**B**) transfected with three different amounts of miR-26a-5p mimics (2, 5 and 8 μg) with negative controls. Abbreviations: UC: Untreated control; TR: Transfection reagent; Ns: no significance.

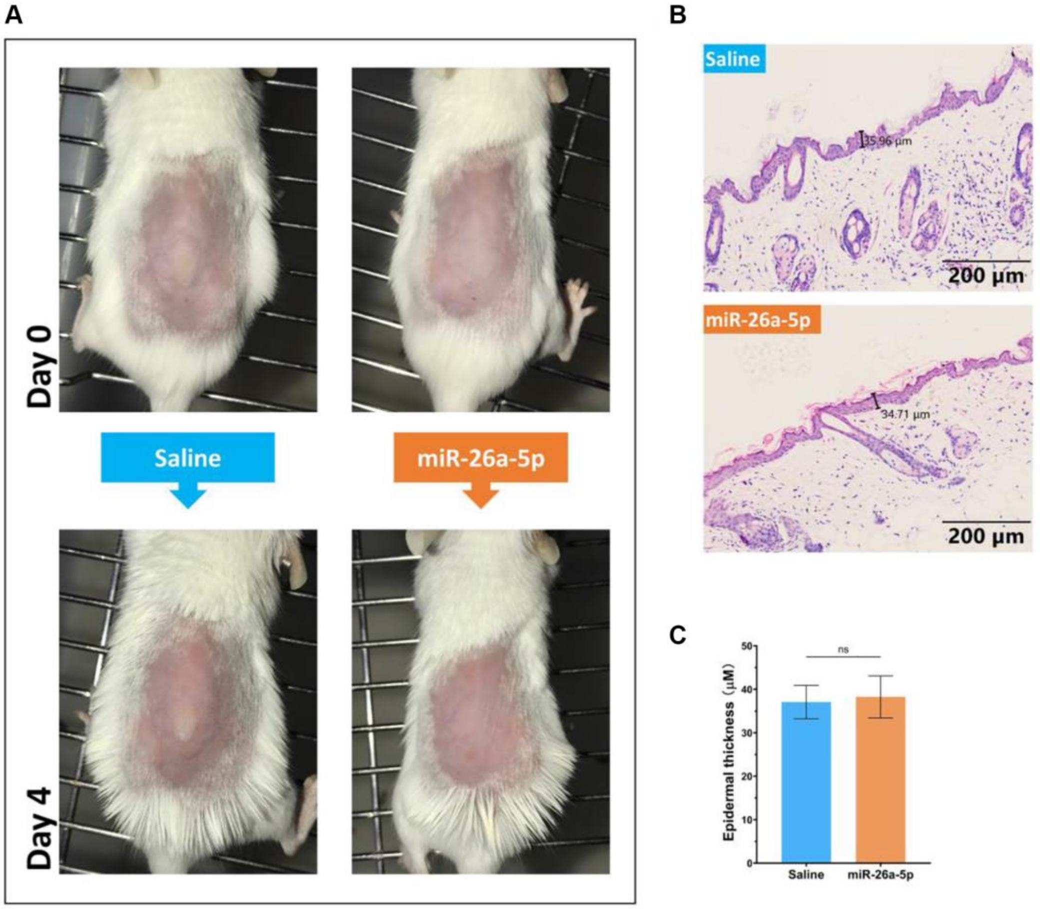

Supplementary Figure 2. Experiment on the safety of miR-26a-5p mimics to normal mouse skin without IMQ induction. (**A**) Skin appearance comparison between mice injected with Saline and 10 μg miR-26a-5p. (**B**) Graphs of H&E-stained skin tissue sections and (**C**) Comparison of thickness of the skin epidermal layers. Abbreviation: Ns: no significance.
